# The Influence of Gastric Microbiota and Probiotics in *Helicobacter pylori* Infection and Associated Diseases

**DOI:** 10.3390/biomedicines13010061

**Published:** 2024-12-30

**Authors:** Jagriti Verma, Md Tanveer Anwar, Bodo Linz, Steffen Backert, Suneesh Kumar Pachathundikandi

**Affiliations:** 1 Department of Environmental Microbiology, School of Earth and Environmental Sciences, Babasaheb Bhimrao Ambedkar University, Vidya Vihar, Raebareli Road, Lucknow 226025, India; 2 Chair of Microbiology, Department of Biology, Friedrich Alexander University Erlangen-Nürnberg, Staudtstr. 5, 91058 Erlangen, Germany

**Keywords:** *H. pylori*, gastric microbiota, probiotics, peptic ulcer disease, gastric cancer, MALT lymphoma

## Abstract

The role of microbiota in human health and disease is becoming increasingly clear as a result of modern microbiome studies in recent decades. The gastrointestinal tract is the major habitat for microbiota in the human body. This microbiota comprises several trillion microorganisms, which is equivalent to almost ten times the total number of cells of the human host. *Helicobacter pylori* is a known pathogen that colonizes the gastric mucosa of almost half of the world population. *H. pylori* is associated with several gastric diseases, including gastric cancer (GC) development. However, the impact of the gastric microbiota in the colonization, chronic infection, and pathogenesis is still not fully understood. Several studies have documented qualitative and quantitative changes in the microbiota’s composition in the presence or absence of this pathogen. Among the diverse microflora in the stomach, the Firmicutes represent the most notable. Bacteria such as *Prevotella* sp., *Clostridium* sp., *Lactobacillus* sp., and *Veillonella* sp. were frequently found in the healthy human stomach. In contrast, *H*.*pylori* is very dominant during chronic gastritis, increasing the proportion of Proteobacteria in the total microbiota to almost 80%, with decreasing relative proportions of Firmicutes. Likewise, *H. pylori* and *Streptococcus* are the most abundant bacteria during peptic ulcer disease. While the development of *H. pylori*-associated intestinal metaplasia is accompanied by an increase in Bacteroides, the stomachs of GC patients are dominated by Firmicutes such as *Lactobacillus* and *Veillonella*, constituting up to 40% of the total microbiota, and by Bacteroidetes such as *Prevotella*, whereas the numbers of *H. pylori* are decreasing. This review focuses on some of the consequences of changes in the gastric microbiota and the function of probiotics to modulate *H. pylori* infection and dysbiosis in general.

## 1. Introduction

The study of the microbiota in humans and animals is increasingly being used to understand the influence on several clinical conditions [1]. The microbiota community of the intestine is one of the most studied entities in this case. Intestinal microbiota has been implicated in several conditions, which affect the gastro-intestinal (GI) area to several internal organs [2,3]. Therefore, the impact of microbiota communities on human physiological and clinical conditions is of greater value in current research. Gastric tissue is generally considered as the most sterile area in the GI tract due to the low pH of the gastric juice, thick mucus layer, peristalsis, and continuous emptying of the stomach [4]. However, the well-known gastric pathogen *Helicobacter pylori* is able to colonize the gastric mucosal epithelium. *H. pylori* produce the enzyme urease that hydrolyses urea to ammonia and CO_2_; thus, it neutralizes the acidic pH in the bacterial cytoplasm and in the immediate gastric environment. In addition, unipolar sheathed flagella allow *H. pylori* to swim through the thick mucus layer and attach to gastric epithelial cells [4,5]. *H. pylori* colonization of the stomach is often associated with the development of gastritis, peptic ulcer disease (PUD), gastric cancer (GC), and mucosa-associated lymphoid tissue (MALT) lymphoma. *H. pylori* type I strains that contain the cytotoxin-associated gene (*cag*) pathogenicity island (*cag*PAI) are more virulent compared to the *cag*PAI-negative type II isolates. The *cag*PAI is thought to be acquired by horizontal DNA transfer from a currently unknown source. It encodes a type IV secretion system (T4SS) that mediates injection of the CagA protein and ADP-heptose into gastric epithelial cells [6]. T4SS-mediated delivery of these effector molecules into the host cells induces high expression of cytokines and chemokines, especially interleukin 8 (IL-8) that attracts neutrophils to cause inflammation. Once transferred, CagA can be tyrosine-phosphorylated by the Src and Abl kinases, and both non-phosphorylated and phosphorylated CagA forms interfere with the cellular actin machinery, cell polarity, and other signaling leading to oncogenic changes [6,7]. Transgenic expression of CagA in the mouse gastric epithelium was sufficient to induce carcinogenesis and confirmed the oncogene status of *cagA* [8]. The drosophila and zebrafish models were also used to demonstrate increased epithelial proliferation and/or the development of hyperplasia/adenocarcinoma due to the transgenic expression of CagA [9,10]. Another major *H. pylori* virulence factor, the vacuolating cytotoxin (VacA), is expressed as 135–140 kDa (p140) pro-toxin and further processed to p88 (mature toxin) and finally into p55 and p33 subunits for triggering cellular vacuolation, apoptosis, and also inhibition of T cell proliferation [11]. Although *H. pylori* virulence determinants CagA and VacA exert the most prominent cellular changes and damage during the infection, *H. pylori* factors such as lipopolysaccharide (LPS), ADP-heptose, peptidoglycan (PGN), cholesterol glucosides, neutrophil activating protein A (NapA), γ-glutamyl transferase (GGT), urease, serine protease HtrA, and a repertoire of adhesion proteins are also playing important roles in the colonization, adhesion, survival and chronicity of infection [12,13]. Of those, serine protease HtrA destroys the epithelial layer by cleavage of cell-to-cell junction proteins, which allows paracellular migration of *H. pylori* to the basolateral cell membranes and subsequent enhanced injection of CagA into the epithelial cells [14,15].

More than half of the world population is estimated to be colonized with *H. pylori*. However, some of the *H. pylori*-infected population develop pathological conditions such as PUD, GC, and MALT lymphoma in 10–20% of total infected individuals, which largely depends on the inflammatory responses [4]. The exact mechanism for development of *H. pylori*-associated severe diseases is yet to be fully understood, but *H. pylori*-induced DNA damage by introduction of double-strand breaks in host chromosomes appears to be a key factor for the development of severe disease [16]. There are many questions that need to be answered. It is not well understood how the different gastric microbiota affects *H. pylori* colonization, growth, chronicity of infection, and pathogenesis. One of the earliest studies identified that some *Lactobacillus* sp. colonized the gastric tissue [17]. Probiotics such as lactic acid bacteria (LAB) are described as live microorganisms that, when given in sufficient amount, have a positive impact on the host’s health [18,19]. In addition to their antagonistic qualities, their probiotic capacity to survive in low pH and high bile salt concentrations as well as their capacity to colonize gastrointestinal surfaces make them one of the most promising and prospective research areas. Probiotic antagonistic action against *H. pylori* has been the subject of several investigations, with encouraging findings in terms of decreasing side effects of antibiotic therapy, enhancing *H. pylori* eradication and minimizing cell damage. Some of the tested probiotic strains can help in reducing *H. pylori* colonization and associated diseases as well as in treatment and mitigation of adverse effects [18,20]. Next-generation sequencing and metagenomics then propelled this field forward, and several compelling data were published based on sequence analysis and culture [21]. Here, we discuss the role and function of the gastric microbiota and probiotics in a healthy stomach and during *H. pylori* infection, including *H. pylori*-associated diseases such as GC.

## 2. Major Factors Involved in *H. pylori* Infection and Pathogenesis

In the pathogenesis of *H. pylori* infection, the host immune responses and subsequent inflammation increase the production of gastric acid by parietal cells and atrophy of the gastric tissues, which causes various pathologies [22]. *H. pylori* usually persist in the gastric pit area if not eradicated by therapy. The chronic inflammation leads to gastric atrophy, metaplasia, and eventually to GC [23]. The elevated acid production by the gastric glands that is triggered by *H. pylori* infection may lead to duodenal ulcer, whereas gastric atrophy is mainly related to the pathogenesis of gastric ulcer or cancer [23]. The gastric inflammation is largely driven by the presence of type-I strains harboring the *cag*PAI and its T4SS. The T4SS-mediated delivery of effector protein CagA and of ADP-heptose into the infected host cells triggers inflammatory signaling [24]. Moreover, T4SS interaction with multiple integrins and TLR5 can also aggravate signaling pathways, which ultimately lead to the expression of pro-inflammatory factors [25,26]. Moreover, multiple monomeric VacA molecules arrange to form membrane channels in the host epithelial cells. Different proteins such as receptor protein tyrosine phosphatase (RPTP) α/β and low-density lipoprotein receptor related protein 1 (LRP1) are described as the main receptors of VacA in epithelial cells [27]. However, in human T-cells, VacA enters through interaction with the receptor protein CD18 or the beta 2 integrin subunit of leukocyte functional antigen 1 (LFA1) [28]. VacA signaling causes various effects such as gastric epithelial cell vacuolization as well as apoptotic and autophagic cell death and inflammation, which may lead to gastric ulcer or cancer [5]. These two virulence factors (CagA and VacA) in tandem determine the majority of the deleterious effects of *H. pylori* infection. Moreover, the other major *H. pylori* factors involved in inflammation are LPS and the LPS metabolite ADP-heptose [29,30]. *H. pylori* LPS is reported to be activating signaling through TLR2, TLR4, and TLR10, which is partially dependent on the acylation and phosphorylation status of Lipid A component [31]. *H. pylori*-derived ADP-heptose induces another inflammatory pathway through activation of alpha protein kinase-1 (ALPK1) and subsequent phosphorylation of tumor necrosis factor (TNF) receptor-associated factor (TRAF)-interacting protein with a forkhead-associated (FHA) domain (TIFA) to activate the canonical pro-inflammatory NF-κB pathway for the expression of IL-8 in the initial hours of infection [30,32]. In addition to the above factors, the pathogenesis of *H. pylori* infection-associated diseases is also closely linked to environmental parameters as well as to host and bacterial genetic factors. Specific polymorphisms in human cytokine and pattern recognition receptor (PRR) genes, GI microflora, and environmental factors such as smoking and alcohol consumption are other major factors influencing the pathogenesis of *H. pylori* [33].

Phylogenetic studies revealed that *H. pylori* has co-evolved with its human host since the origin of humans, at least for 100,000 years or likely longer [34,35]. *H. pylori* has been diversifying with human populations, and extant strains are classified into eight major bacterial populations that were named after their predominant geographic sources: hpAfrica1, hpAfrica2, hpNEAfrica, hpEurope, hpEastAsia, hpAsia2, hpNorthAsia, and hpSahul [36]. *H. pylori* strains exhibit substantial variation in the CagA protein, particularly in repeated Glu-Pro-Ile-Tyr-Ala protein sequences, the so-called EPIYA motifs. There are four distinct motifs, EPIYA-A, EPIYA-B, EPIYA-C, and EPIYA-D, that are characterized by specific flanking amino acid sequences. Strains isolated from East Asia (*H. pylori* population hpEastAsia) express CagA with EPIYA-A, EPIYA-B, and EPIYA-D motifs, while strains from Western countries contain the EPIYA-A, EPIYA-B, and EPIYA-C motifs [37,38]. In East Asian countries, almost all *H. pylori* isolates possess the *cag*PAI T4SS, but in Western countries about 40% of the strains do not contain this genotype. The presence of the *cag*PAI is usually accompanied by the cytotoxic *vacA s1/m1* or *s1/m2* alleles. In contrast, *cag*PAI-negative *H. pylori* commonly express the non-toxic *s2/m2* allele VacA variant with a stretch of hydrophilic amino acids in the *s2* signal peptide region that prevents the formation of a pore across the host membrane. Thus, *vacA s2/m2 cag*PAI-negative strains are relatively benign in their interaction with the gastric epithelium, but infection with *vacA s1/m1 cag*PAI-positive strains is considered a risk factor for the development of gastric disease [39]. Thus, GC rates are highest in East Asian countries, but comparatively low in African countries [40]. Phosphorylated CagA interacts with Src homology-2 (SH2) domain containing human proteins like CSK, GRB2, and SHP2 to change cell proliferation and differentiation inside the gastric epithelial cells [41]. The above interactions of CagA change the cytoskeletal pattern and induce increased pro-inflammatory cytokine (IL-8) secretion via the NF-κB pathway [42]. As stated above, inside the host gastric epithelial cells, CagA functions as an oncoprotein and activates carcinogenic signal transduction pathways. CagA protein triggers inflammation, changes cell shape and polarity, averts cell death by inhibition of apoptosis, and leads to cancer and ulcer in the infected tissue [11]. On the other hand, VacA modulates the cellular machinery and stimulates apoptosis and exhibits some antagonistic effects [43,44]. Phosphorylated CagA prevents VacA from reaching its destination within the cell, while non-phosphorylated CagA directly mitigates the detrimental effect of VacA on mitochondria, without impeding VacA movement within the cell [11]. The expression of programmed cell death-1 (PD1) protein in infected gastric epithelial cells positively correlated with *vacA m1/m2* in patients with gastritis and ulcers, but inversely correlated with *vacA s1/m1*. Likewise, a negative correlation was also observed with PD1 Ligand-1 (PD1L1) expression [45].

In addition to the presence of the *cag*PAI and the *vacA s1/m1* allele, three SNPs in the serine protease gene *htrA* were shown to correlate with risk of severe disease development, particularly of GC [15]. Detailed analyses of one of those SNPs, a TCA/TTA polymorphism that resulted in a serine (S) to leucine (L) amino acid change at protein position 171, revealed the underlying mechanisms. The 171L-type HtrA increased structural stability of HtrA trimers, the proteolytically active version of this serine protease, and thus amplified the protease function of the enzyme [15,46]. This resulted in increased destruction of the epithelial barrier by cleavage of epithelial cell-to-cell junction proteins E-cadherin, occludin, and claudin-8. Cleavage of E-cadherin released β-catenin from the E-cadherin/β-catenin complex, which led to its accumulation in the nucleus where it increased cell proliferation. Strains possessing 171L-HtrA further enhanced CagA injection into epithelial host cells, induced more severe inflammation, and triggered double strand breaks in host chromosomes [15]. All these effects greatly enhance the risk of GC development. The interplay of these major virulence factors shapes the *H. pylori* survival, persistence and pathogenesis of gastric diseases in some of the colonized hosts. The presence and absence of *H. pylori* T4SS, CagA, VacA, and HtrA can impact the inflammatory signaling, cell polarity, and integrity, which ultimately reduce microbiota colonization and population diversity. Therefore, the influence of microbiota and probiotics in the pathogenic mechanisms of *H. pylori* must be considered from the perspective of the associated diseases.

## 3. Major Microbiota Patterns Observed in the Gastric Area

**Figure 1:**
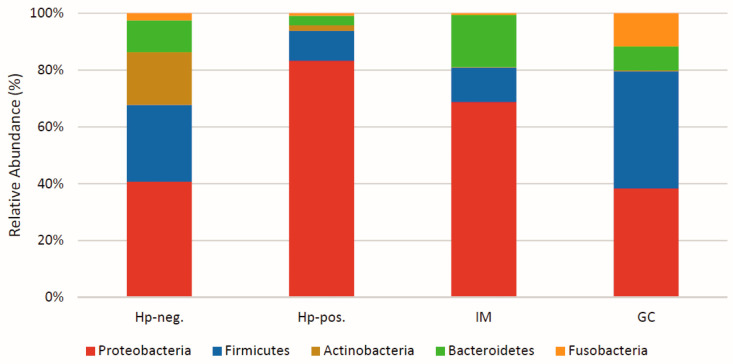
Changes in the gastric microbiota following *Helicobacter pylori* infection. Schematic representation of the predominant phyla of the gastric microbiota in *H. pylori*-negative (Hp-neg.) and in *H. pylori*-positive individuals (Hp-pos.) and in individuals with intestinal metaplasia (IM) or with GC.

The number of microorganisms inhabiting the GI tract was estimated to be over 100 trillion, which exceeds the total number of human cells in the body about 10 times [47]. The human microbiome project has identified 2172 species from 12 different phyla [48]. The major bacterial and archaeal phyla that were frequently observed in the gut are Proteobacteria, Firmicutes, Actinobacteria, Fusobacteria, Verrucomicrobia, Bacteroidetes, Cyanobacteria, Saccharibacteria, Spirochaetes, and Euryarchaeota [49,50,51,52,53]. Firmicutes, Proteobacteria, Actinobacteria, and Bacteroidetes are the most dominant phyla in the adult gut, constituting about 90% of the total microbiota (Figure 1) [54,55,56,57,58]. The microflora of these phyla plays an important role in the intestinal metabolism. The high microbial diversity ensures that metabolic function is not impaired by changes in microbiota composition due to microbial redundancy [1]. The fungal microbiota mainly belongs to the genera *Candida*, *Saccharomyces*, *Malassezia*, and *Cladosporium* [59]. The diversity of the microbiota is high in adults when compared to children, but decreases in the elderly and during dysbiosis that occurs due to several inflammatory conditions [54,60]. The total bacterial numbers and the composition of microbiota depend on the position in the host GI tract and various factors such as pH, bile acids, digestive enzymes, IgA, antimicrobial peptides (AMPs), miRNAs, and other intestinal/environmental factors [61]. The relationship between the normal gastric microflora of the stomach and the host is mutualistic and beneficial for both [62]. Members of the *Lactobacillus* genus such as *L. gasseri*, *L. fermentum*, and *L. rhamnosus* usually present in the stomach are highly important for a healthy human gut [4]. Additionally, using 16S rRNA sequencing-based techniques, early studies of the gastric microflora in healthy individuals identified the presence of *Streptococcus*, *Prevotella*, *Veillonella*, *Gamella*, *Neisseria*, *Fusobacterium*, *Pseudomonas*, etc. (Table 1) [63]. This resident microbiota has important functions. For example, *Streptococcus* species such as *S. salivarus* and *S. mitis* inhibit the growth of *H. pylori* [4]. In mouse models of *H. pylori* infection, the presence of *Clostridium* sp. interferes with *H. pylori*-mediated recruitment of CD4^+^ T-cells to the stomach mucosa, which shows the impact of microbiota composition in gastritis [64]. In contrast, abundance of several *Clostridium* species may increase the malicious response to *H. pylori* infection, the onset of pangastritis, atrophic gastritis, and GC [56,65]. A comparative study of the gastric microbiota in different *H. pylori*-infected and non-infected samples was able to differentiate the bacterial communities. The study also showed that age, growth, and gender do not represent main factors driving the microbial composition in the gastric region [66]. Together, this field of research is still in the early stages and revealed various unsolved questions. We still do not know the exact microflora that exhibits beneficial effects such as reducing the growth of *H. pylori* in the gastric mucosa *in vivo*.

## 4. Gastric Microbiota Dynamics in *H. pylori* Colonization and Associated Conditions

The composition of the gastric microbiota differs from person to person, region to region, and ethnicity to ethnicity and is influenced by a variety of factors such as diet, host genetics, health, age, and environmental conditions [58,70,71,72,73]. The microbiota usually consists of numerous taxa of the phyla Proteobacteria, Firmicutes, Actinobacteria, Bacteroides, and Fusobacteria in varying abundance (Figure 1). *H. pylori*-negative individuals usually have a microbiota that is more complex and very diverse compared to *H. pylori*-positive individuals [2,21,55,56,57,58,74,75]. During *H. pylori* infection, which triggers the development of chronic gastritis, the microbiota is heavily dominated by Proteobacteria (Figure 1). As a result, *H. pylori* infection lowers the overall bacterial diversity (α-diversity), both the diversity and distribution of taxa. In later stages of disease, from intestinal metaplasia to adenocarcinoma of the stomach, the presence of *H. pylori* is greatly reduced and bacteria of other genera are enriched [2,21,55,56,57,58,74,75]. Interestingly, the later stages of the disease are often accompanied by changes in gastric pH due to the decrease in numbers of acid-producing parietal cells, which allows the colonization and survival of bacteria from the oral cavity (e.g., *Neisseria*) as well as intestinal bacteria (Figure 1), thus enhancing the *H. pylori* infection-associated dysbiosis. The invading intestinal bacteria include *Escherichia* and *Burkholderia* (Proteobacteria); *Lachnospiraceae*, *Lactobacillus*, *Streptococcus,* and *Veillonella* (all Firmicutes); and *Prevotella* (Bacteroidetes) [2,56,58,75,76,77].

By bacterial culture, *Clostridium* sp., *Lactobacillus* sp., and *Veillonella* sp. have been frequently observed in the stomach of healthy volunteers, but 80% of the microflora of the stomach is currently not cultivable, including *Leptotrichia* sp., *Shewanella* sp., *Lachnospiraceae* sp., *Porphyromonas* sp., etc., which were identified by 16S rRNA sequencing [78,79,80]. In the human stomach, *H. pylori* co-exists with several microbiota, which initiates substantial crosstalk such as secretion of cecropin-like antibacterial peptides that have pro-inflammatory effects and cause acidosis [81]. *H. pylori* growth was inhibited by *Lactobacillus* species such as *L. johnsonii*, *L. murinus*, and *L. reuteri* *in vitro* [82]. A strain of *L. reuteri* that was isolated from gastric juice showed an antimicrobial effect against *H. pylori* with effective inhibition in the acidic environment of the stomach [83]. In some adverse conditions, the presence of *Streptococcus* has been observed as a perfect companion during development of PUD [69]. *H. pylori* presence is capable of inducing pro-carcinogenic inflammation signaling with the help of bacterial products [84]. Patients with GC were shown to have microbial flora such as *Lactobacillus*, *Streptococcus* (mostly *S. mitis* and *S. parasanguinis*), *Prevotella*, and *Veillonella* [80]. A high abundance of *Pseudomonas* was found among the microflora of patients with neoplastic lesions compared to patients with gastritis [85]. Moreover, *Pseudomonas aeruginosa* and *Staphylococcus aureus* were reported to be present in patients with gastritis and PUD with or without co-infection of *H. pylori* [86]. Further, *Burkholderia pseudomallei* colonization was reported in gastric samples along with *H. pylori*, which may exacerbate chronic and acute infections [87]. In contrast, *S. mitis*, a commensal bacterium, inhibited the development of *H. pylori* and altered its morphology from spiral to the coccoid form [88]. The gastric microbiota has several antibacterial and probiotic properties that might be lost due to treatment of stomach diseases, whereas variability has occurred in the beneficial microflora during the infection of *H. pylori* (Table 2). Besides changes in the gastric microbiota, *H. pylori*-mediated hypochlorhydria and hypergastrinemia also affected the microbiota in the large intestine by changing the abundance of *E. coli*, *Enterococcus*, *Bacteroides*, *Prevotella,* and *Akkermansia* [53,69,89]. Therefore, several researchers assume that other unknown gastric microbiota members are also helping the survival of *H. pylori* and might be supporting the pathogenesis of chronic stomach diseases.

## 5. The Effect of Resident Gastric Microbiota and Probiotics on *H. pylori*-Mediated Inflammation

**Figure 2:**
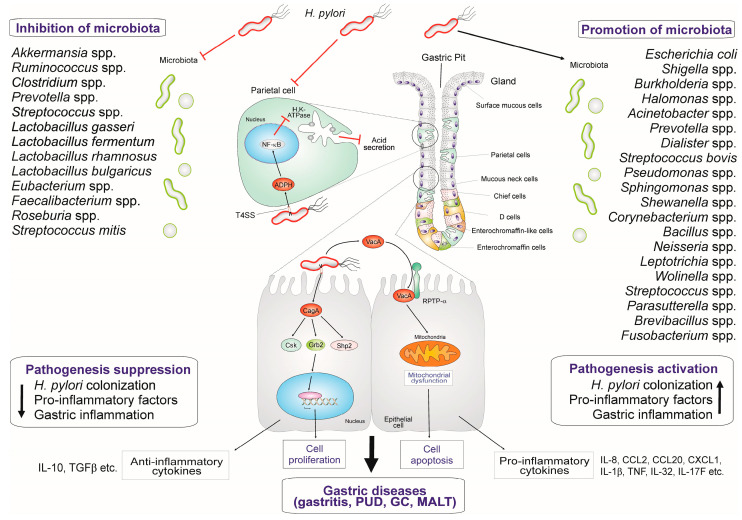
*Helicobacter pylori* infection promoting and inhibiting major microbiota groups in pathogenesis. The dynamics of microbiota composition during *H. pylori* infection might be involved in the associated pathologies. *H. pylori* is a critical factor affecting the microbiota diversity in the gastric mucosa. In the context of an *H. pylori* infection, gastric microbiota groups can be inhibited or promoted. As per the available evidence, these two microbiota groups are functionally different in supporting or preventing the growth of *H. pylori*, immune responses, and pathogenesis of the associated diseases. Thus, the survival and successful colonization of *H. pylori* leads to dysbiosis, which favors persistence of infection.

Several beneficial microbial communities present inside the gastric region are able to create a healthy gastric environment and also challenge the pathogenic microorganisms like *H. pylori* [69]. Once *H. pylori* colonization is established in the gastric mucosa, the host immune responses mount an inflammatory reaction through recruitment of various immune cells to the site of infection. These immune reactions are detrimental to the normal gastric microbiota, and thus may lead to dysbiosis (Figure 2). Therefore, the presence of *H. pylori* causes a number of problems for the gastric immunology [81]. The cultivable isolates from gastric microbiota include mainly bacteria of the four genera *Propionibacterium*, *Lactobacillus*, *Streptococcus,* and *Staphylococcus*. Analysis of gastric microbiota patterns in dyspeptic children showed decreased microbial diversity in the *H. pylori*-infected group when compared to *H. pylori*-negative and control groups. The *H. pylori*-infected children displayed significantly reduced diversity and abundance in six phyla (Actinobacteria, Bacteroidetes, Firmicutes, Fusobacteria, Gemmatimonadetes, and Verrucomicrobia) and eight genera (*Achromobacter*, *Devosia*, *Halomonas*, *Mycobacterium*, *Pseudomonas*, *Serratia*, *Sphingopyxis,* and *Stenotrophomonas*) [91]. The gastric microbiota pattern in children and adults seems to be similar, but *H. pylori*-infected children were found to have more diverse microbiota with smaller abundance of Firmicutes and larger abundance of non-*Helicobacter* Proteobacteria and other taxonomic groups as compared to adults [92]. There is a negative correlation between the load of *H. pylori* and the gastric microbial diversity as the load of *H. pylori* decreases the α-diversity [90]. In children with *H. pylori* infection, there is an increase in CD4^+^ T-cells and macrophages as compared to non-infected children. RNAseq revealed that *H. pylori*-infected children had elevated mRNA expression of *FOXP3*, *TGFB1*, *IL10*, and *IL17* in the stomach [91]. Opportunistic pathogens like *P. acnes* were found to be associated with corpus-dominant lymphocytic gastritis. This bacterium produces small chain fatty acids (SCFAs) such as propionate and butyrate that induces NKG2D-NKG2DL (natural killer group 2 member D)-mediated anti-tumor response and IL-15 expression [93]. However, *H. pylori* infection was found to down-regulate both NKG2D-NKG2DL and IL-15 expression [93]. Therefore, the function of *P. acne*-associated corpus-dominant lymphocytic gastritis in preventing *H. pylori*-associated GC must be investigated further. In addition, galactin-3 is implicated in the Th17 differentiation and microbiota balance in the oral cavity and preventing mucosal infection [94]. The galectin-3 expression is upregulated in the gastric epithelium during *H. pylori* infection [95]. *H. pylori* type-I strains are mainly responsible for the decrease in the gastric microbiota diversity [96]. *H. pylori* strains that express the *dup*A gene are linked to higher risk for erosive gastritis than GC and disappearance of *Streptococcus* and *Prevotella* [97]. Interestingly, isogenic *H. pylori* infection of mice from two different providers, Taconic Sciences (Tac) and the Jackson Laboratory (Jax), have shown different colonization densities, histopathology, and immune responses [98]. The gastric mucosal metaplasia and Th1 immune response associated IgG2c levels were higher in Tac mice compared to Jax mice; therefore, Jax mice showed the highest *H. pylori* colonization [98]. The microbiota communities were different in the stomach and colon of Tac and Jax groups of mice. However, *H. pylori* infection altered the stomach and colon microbiota in the Jax group, but only the composition of the colon microbiota in the Tac group [98]. Interestingly, *Bifidobacterium bifidum* strain BF1 improved the symptoms of gastritis during *H. pylori* infection by suppressing the gene expression that was triggered by *H. pylori*-induced NF-κB signaling, especially the expression of IL-8 [99]. In this context, it can be hypothesized that stomach dysbiosis is an important scenario involved in *H. pylori* colonization and survival. Thus, the gastric microbiome may undergo alterations during prolonged *H. pylori* infection, which is associated with a shift in gastric physiology including modification of innate immune responses, reduced gastric acidity, and adjustment in nutrient accessibility [92].

Several experiments provided evidence that probiotic bacteria such as *L. casei*, *L. paracasei*, *L. acidophilus*, *B. lactis*, and *S. thermophilus* can inhibit the growth of *H. pylori* and kill *H. pylori* *in vitro*. These tested strains showed both bacteriostatic and bactericidal effects against the pathogen [100]. Also, *L. acidophilus* strain ATCC4356 and *L. rhamnosus* strain PTCC1607 showed inhibitory effects on the growth of *H. pylori* under laboratory conditions and were found to hinder the attachment of *H. pylori* to gastric epithelial cells. The major reported effects of probiotics on *H. pylori* infection are listed in Table 3. Furthermore, *L. acidophilus* was found to stimulate macrophages of the U937 lineage to produce interferon-γ (IFNγ), which suggests that *L. acidophilus* bacteria are able to modulate immune responses that protect against *H. pylori* infection [101]. When *H. pylori*-infected mice were treated with *L. fermentum* strain P2, *L. casei* L21, *L. rhamnosus* JB3, or a mixture of these strains, *H. pylori vac*A gene expression was decreased, which reduced specific immune responses in the stomach area. In addition, treatment with LAB resulted in restoring the amount of immune-related fatty acids that are normally reduced during *H. pylori* infection [102]. *L. plantarum* was found to inhibit the growth of *H. pylori* in the gastric mucosa and also reduced the expression of *AKT*, a crucial host gene controlling cell proliferation and other responses. The presence of *L. plantarum* triggered an increase in cell apoptosis and gene expression of TLR4, PTEN, and Bax [103]. *L. gasseri* strain ATCC 33323 proved to be another effective probiotic in reducing *H. pylori*-mediated inflammation by decreasing the expression of β-catenin, integrin-α5β1, and IL-8 in stomach cells during *H. pylori* infection [104]. *L. salivarius* and *L. rhamnosus* were reported to restore the presence of anti-inflammatory bacteria that were reduced by *H. pylori*. These lactobacilli also down-regulated the pro-inflammatory signaling in *H. pylori*-infected cells, including NF-κB, TNF, and IL-17 [105]. *Parabacteroides goldsteinii* strain MTS01 was demonstrated to modulate gut microbiota, to mitigate the harmful effect of *H. pylori* virulence factors VacA and CagA, and to alleviate *H. pylori*-induced inflammation [106]. The neuraminidase activity of the yeast *S. boulardii* was found to remove sialic acid residues from the cell surface of the epithelium in the duodenal region that act as binding sites for *H. pylori* and hence blocks the ability of *H. pylori* to adhere to the cells [107]. Evidently, LAB are effective in retarding *H. pylori* growth, decreasing its urease activity, hindering its flagella-mediated motility, and also in reducing the inflammation by decreasing its ability to induce IL-8 secretion in human gastric epithelial cells [108]. *L. delbrueckii* subsp. *bulgaricus* strains produce bacteriocin-like inhibitory substances and exhibited strong anti-*H. pylori* effects [109,110]. In addition, *L. salivarius* B101, *L. rhamnosus* B103, and *L. plantarum* XB7 were found to downregulate IL-8 expression and secretion by blocking NF-κB and MAPK signaling [111,112]. Several clinical trials were conducted to identify the efficacy of probiotics in preventing or controlling *H. pylori* infection and associated diseases. The results of those clinical trials were summarized in a recent meta-analysis. This comprehensive review of 28 meta-analyses from 534 randomized clinical trials showed that supplementation with probiotics improved the eradication rate of *H. pylori* with less side effects [113]. Together, the above data demonstrate that *H. pylori* infection results in drastic changes in the α-diversity of the gastric microbiota, which in turn causes dysbiosis, an increase in stomach pH, and a host immune response that benefits persistent *H. pylori* infection. These negative effects can be partially mitigated by the application of several probiotic bacteria, particularly LAB.

## 6. Gastric Microbiota Functions in the *H. pylori*-Associated GC Pathogenesis

Microbes are involved in the induction and maintenance of carcinogenesis by various pathways such as stimulation of inflammation, increasing cell proliferation, stem cell physiology dysregulation, and production of various metabolites [114]. In order to explore the reason behind the occurrence of GC even after eradication of *H. pylori*, the dysbiosis after eradication of *H. pylori* was analyzed in detail [115]. This study found that there was an abundance of bacteria from 29 different genera including *Fusobacterium* and *Neisseria* species that were frequently identified in samples from GC patients. Metabolites from *Fusobacterium* were found to be genotoxic, and presence of these bacteria was linked to inflammatory reactions and higher tumor mutation burden [115]. An analysis of the microbiome shifts during the progression of gastric carcinoma revealed that around forty taxonomical units are differently abundant. Some of the major genera are *Bifidobacterium*, *Streptococcus anginosus*, *Leptotrichia*, *Fusobacterium*, *Prevotella*, *Actinobacillus parahaemolyticus*, *Selenomonas*, *Lactobacillus mucosae*, *Veillonella*, *Dialister*, *Lachnospira*, *Parvimonas*, and *Clostridium* [116]. *H. pylori* massively alters the properties of all beneficial microbial flora in the gastric region. When the stages of cancer progress during GC development, the concentration of *H. pylori* decreases, and the stomach gets colonized by genera such as *Lactobacillus*, *Prevotella*, *Streptococcus*, *Achromobacter*, *Citrobacter*, *Clostridium*, *Rhodococcus*, and *Phyllobacterium* [117]. The influx of other bacterial genera is in part associated with changes in gastric acidity as the pH is rising due to damage of acid producing parietal cells. In contrast, samples from patients with *H. pylori*-negative gastritis were enriched with bacteria of the genera *Dialister*, *Paludibacter*, *Streptococcus*, *Treponema,* and *Haemophilus* and of the phylum Actinobacteria [117]. In contrast, bacteria of the phylum *Saccharibacteria* (TM7) were more prevalent in the *H*. *pylori*-positive group than the non-*H. pylori* group. At the genus level, *Bifidobacterium* and *Bacteroidetes* were less prevalent in the *H. pylori*-positive group than in the non-*H. pylori* group [67]. *H. pylori* is the main causative factor for initiating GC; additionally, non-*H. pylori* microflora have been described mainly at the final steps of the carcinogenesis [76]. The composition of the gastric microbiota varies among individuals with chronic gastritis, precancerous lesions, and GC (Figure 1) [118]. Some studies have reported that individuals who have a fair proportion of *Bifidobacterium* in their lower GI tract are generally protected against severe problems from *H. pylori* infection. Typically, those individuals do not develop GC or PUD, indicating that these bacteria may act as a probiotic [67]. Bacteria such as *Streptococci* have been seen to grow more frequently in the gastric tumor region compared with the normal region of the stomach [80]. Gastric atrophic changes during *H. pylori* infection were accompanied by dominating *Streptococcus* sp., *Lactobacillus* sp., *Veillonella* sp., and *Prevotella* sp. [4]. *S. bovis* is associated with colon cancer, and the antigens of this bacterium have been reported to initiate cancer in mice. *H. pylori* CagA and *S. bovis* cell wall antigens increase expression of inflammatory mediators known to accompany carcinogenesis, such as IL-8, prostaglandin E2 (PGE2), and cyclooxygenase (COX2) [80]. Some researchers assumed that selected microbes may play an important role in combination with *H. pylori* to cause GC, but there is currently no clear consensus for a specific microflora that is associated with *H. pylori* and GC development. Further studies are required on this unresolved issue.

## 7. *H. pylori*-Associated PUD Pathogenesis and Microbiota

The lifetime occurrence of PUD in *H. pylori*-colonized individuals is one in ten cases [119]. Approximately 90% of the duodenal ulcers and 70–90% of gastric ulcers in patients are directly related to *H. pylori* infection. A meta-analysis has found that there is a strong correlation between *H. pylori* and chronic gastritis, while the impact of non-*H. pylori* bacteria is not very clear [120]. In PUD, beneficial microflora such as members of the *Lactobacillus* group, *Clostridium leptum* subgroup, and *Enterobacteriaceae* are suppressed in the presence of *H. pylori*, and their distribution and re-colonization in ulcer patients may be affected by gender [121]. Bacteria isolated from *H. pylori*-positive patients with PUD were very different from patients with non-*H. pylori* PUD or from the control group with normal gastric microbiota. During the development of PUD, the abundance and diversity of microbiota gradually decreased, and the *H. pylori* infection became chronic [122]. The microbiota in *H. pylori*-positive patients with PUD that encompasses genera such as *Streptococcus*, *Neisseria*, *Rothia*, and *Staphylococcus* are thought to promote and exacerbate the infection [122].

*H. pylori* infection triggers the influx of IgG into the gastric epithelium, which contributes to the destabilization of the epithelial barrier and allows leakage of the gastric acid into deeper tissue layers and hence leads to the progression of PUD [123]. It has been reported that the patients with *H. pylori* infection exhibited more severe ulceration compared to ulcer patients without *H. pylori* [123]. In the *H. pylori*-infected population, the chance of having PUD is six to ten times higher than in *H. pylori*-negative individuals [124]. Treatment of PUD usually involves lowering acids level in the stomach by acid blockers and eradication of *H. pylori* by antibiotics [125]. In addition, several probiotic bacteria may play a crucial role and show an inhibitory effect on *H. pylori* infection and thus may represent effective tools in controlling *H. pylori*-mediated PUD [100,101]. According to a recent study, there is a link between gut bacteria and the development of gastric and duodenal ulcers. In the development of gastric ulcers, *Clostridium*, *Butyriccocus,* and *Peptococcus* bacteria were harmful, whereas *Lachnospiraceae* UCG004 as well as *Mollicutes* strain RF9 appeared to be beneficial. On the other hand, in case of a duodenal ulcer, bacteria like *Lentisphaeria* and *Negativicutes* were identified as potentially harmful, while *Catenibacterium*, *Escherichia* and *Shigella* are considered as beneficial [126]. The patients with duodenal ulcers were shown to harbor a greater variety of bacteria in their tissue compared to those with gastric ulcers. For example, in addition to *H. pylori*, other bacteria like *Prevotella*, *Neisseria,* and *Streptococcus* were also found in those patients [127]. Though there is currently no evidence of the mechanisms by which the bacteria are involved in PUD, many bacteria like *Lactobacillus* UCG004, *Mollicutes* strain RF9, *Catenibacterium*, *Escherichia*, *Shigella*, *Lactobacillus* UCG008, and *Sutterella* are found to play a protective role during PUD treatment [128]. Hence, the microbiota may be a potential tool for the treatment of PUD in patients.

## 8. Conclusions

*H. pylori* is a gastric pathogen that can modulate the normal GI microbiota for its survival, which leads to dysbiosis and paves the way for the pathogenic effects of the infection. Several studies have been conducted to understand the microbial communities of the gastric area; however, the microbes vary in terms of their presence and ratio in various niches. While *Streptococcus* sp., *S. aureus*, and *P. aeruginosa* were found to be associated with *H. pylori* gastritis or PUD conditions, *Streptococcus*, *Prevotella*, and *Veillonella* appear to be the dominating genera in *H. pylori*-associated GC tissue. Evidently, the microbiota composition changes in the stomach during *H. pylori* infection, partially because of human immune responses, and at later stages due to neutralization of the acidity in the gastric lumen. *H. pylori* growth was hampered by LAB; randomized clinical trials showed that probiotics have the potential to mitigate some of the adverse effects of an *H. pylori* infection by enhancing the efficacy of eradication therapy, reducing the severity of GI symptoms and by helping to restore the microbiome after *H. pylori* eradication. Food-derived prebiotic compounds are another important factor in providing support to the gut microbiota in general. Here, future studies are required to assess the influence of confounding factors such as lifestyle, diet, mainly plant-based diet-derived prebiotics and signaling molecules, the use of over-the-counter antibiotics, and others, on modulating the microbiome and *H. pylori* infection.

## Figures and Tables

**Table 1:** Common gastric microbiota phyla and genera reported in various studies.

S. No.	Phylum	Genus	Function	References
1.	Firmicute	*Lactobacillus* *Peptostreptococcus* *Protococcus* *Streptococcus* *Clostridium* *Eubacterium* *Faecalibacterium* *Roseburia* *Dorea* *Ruminococcus*	Probiotic strain Butyrate producer Green algae Commensal strain Probiotic strain Butyrate producer Butyrate producer Butyrate producer Butyrate producer *Keystone* sp.	[4,51,52,60,62]
2.	Bacteroidetes	*Bacteroides* *Prevotella* *Xylanibacter*	Probiotic strain Probiotic strain Probiotic strain	[4,52,60,62]
3.	Actinobacteria	*Bifidobacterium*	Probiotic strain	[51,52,62,67]
4.	Proteobacteria	*Escherichia coli* *Actinobacter* *Haemophillus* *Serratia* *Neisseria* *Stenotrophomonas* *Burkholderia*	Commensal strain Probiotic strain *Keystone* sp. *Keystone* sp. Commensal strain *Keystone* sp. *Keystone* sp.	[21,62,68]
5.	Verrucomicrobia	*Akkermansia muciniphila*	*Keystone* sp.	[53,69]

**Table 2:** *H. pylori* and connection with gastrointestinal microbiota in associated diseases.

S. No.	Bacterial Genera Present in *H. pylori* Colonization	Location	Disease Connection	References
1.	*Porphyromonas* sp. *Neisseria* sp. *S. coleohominis*	Gastric mucosa	Gastritis	[69]
2.	*Akkermansia* sp.	Gastrointestinal tract	Chronic Gastritis	[53,69]
3.	*Escherichia* *Shigella* *Burkholderia* *Halomonas*	Gastrointestinal region	Gastritis and GC	[21,88]
4.	*Clostridium*	Gastrointestinal tract	Gastritis and GC	[56,65]
5.	*Lactobacillus * *Streptococcus * *Veillonella*	Gastric mucosa	Gastritis and GC	[21,68,80]
6.	*Pseudomonas* *Sphingomonas* *Shewanella* *Corynebacterium* *Bacillus* *Neisseria* *Leptotrichia*	Gastrointestinal tract	GC	[76]
7.	*Prevotella*	Gastrointestinal tract, mouth and vagina	GC	[68,76,80]
8.	*Pseudomonas* sp.	Gastric mucosa	GC	[85]
9.	*Wolinella* sp.	Gastric mucosa	GC	[90]
10.	*S. parasanguinis*	Gastric mucosa	GC	[69]
11.	*Acinetobacter*	Gastrointestinal tract	GC	[76]
12.	*Parasutterella* *Brevibacillus* *Fusobacterium*	Gastric mucosa	GC	[37]
13.	*Oscillospira* *Oscillibacter* *Lachnoclostridium*	Gastrointestinal tract	GC	[37]
14.	*S. bovis*	Gastrointestinal tract	Colon cancer	[80]
15.	*Dialister*	Gastrointestinal tract	GC	[67]
16.	*B. pseudomallei*	Gastric mucosa	Influence the gastric microbiota or opportunistic pathogen	[87,88]
17.	*Oscillospira*	Gastrointestinal tract	*H. pylori*-associated diseases.	[37,67]

**Table 3:** *Helicobacter pylori* infection control in the presence of different probiotics.

S. No.	Name of Probiotic Bacteria/Strains	Effect in Gastric Region in *H. pylori* Infection Condition	References
1.	*L. johnsoni* *L. murinus* *L. reuteri*	*H. pylori* growth inhibition	[62,69]
2.	*L. casei* *L. paracasei* *L. acidophilus* *B. lactis* *S. thermophilus*	Bacteriostatic and bactericidal effects against *H. pylori*	[100]
3	*Bifidobacterium*	Probiotic alone altered the diversity and composition of gastric microbiota without inhibiting effect against *H. pylori*.	[100]
4.	*L. acidophilus* ATCC4356, *L. rhamnosus* PTCC1607	*H. pylori* growth inhibition Prevent *H. pylori* from attaching to the gastric MKN-45 cells Stimulates the production of IFNγ by macrophages (U937 cells)	[101]
5.	*L. fermentum* P2 *L. casei* L21 *L. rhamnosus* JB3	All probiotics reduce *H. pylori* levels, *vacA* gene expression, and specific immunoglobulin levels in stomach. Increased serum level of IFNγ and IL-1β leading to immune response Multi-LAB restores the antioxidant activities suppressed by *H. pylori*, adjust metabolite composition, and increase in essential amino acids important for immune function.	[102]
6.	*L. plantarum*	Inhibits *H. pylori* and GC cell line (AGS). Increase in gene expression of PTEN, Bax and TLR-4. Decrease in AKT gene expression. Increases cell apoptosis.	[103]
7.	*L. gasseri* ATCC 33323	Reduces inflammation caused by *H. pylori*. Reduces the expression of Bcl-2, B- catenin, integrin-α5, integrin B-1, and IL-8 in stomach cells infected with *H. pylori*.	[104]
8.	*L. salivarius* *L. rhamnosus*	Restores the anti- inflammatory bacteria reduced by *H. pylori*. Down regulates the pro-inflammatory signaling including NF-KB, TNF, IL17 in *H. pylori* infected cells.	[105]
9.	*P. goldsteinii* MTS01	Reduces the harmful effect of VacA and cagA of *H. pylori*. Changes the microbiota and lowers the cholesterol level easing the *H. pylori* induced inflammatory reaction to take place.	[106]
10.	*S. boulardii*	Shows neuraminidase activity and removes surface α (2–3)-linked sialic acid (it acts as a ligand for *H. pylori*) on the cell surface of duodenal region.	[107]
11.	LAB	Restricts the growth of *H. pylori* Reduces the urease activity of *H. pylori*. Hinders the *H. pylori* flagella-mediated motility. Reduces the ability of *H. pylori* to induce the pro-inflammatory IL-8 in the human gastric cells.	[108]
12.	*L. delbrueckii* subsp. bulgaricus	Bacteriocin-like inhibitory substance is produced Suppresses the secretion of pro- inflammatory cytokine IL-8 by gastric epithelial cells. Shows strong anti *H. pylori* activity.	[109,110]
13.	*L. salivarius* B101 *L. rhamnosus* B103 *L. plantarum* XB7	Suppresses IL-8 secretion and mRNA expression Inhibits NF-KB activation Suppresses c-Jun activation	[111]
14.	*L. plantarum* ATCC8014	Reduces gastric inflammation and shows anti *H. pylori* effect.	[112]

## Data Availability

No new data were created.

## References

[1] Hou K., Wu Z.-X., Chen X.-Y., Wang J.-Q., Zhang D., Xiao C., Zhu D., Koya J.B., Wei L., Li J. (2022). Microbiota in Health and Diseases. Sig. Transduct. Target. Ther..

[2] Mendes-Rocha M., Pereira-Marques J., Ferreira R.M., Figueiredo C. (2023). Gastric Cancer: The Microbiome Beyond *Helicobacter pylori*. Curr. Top. Microbiol. Immunol..

[3] Engelsberger V., Gerhard M., Mejías-Luque R. (2024). Effects of *Helicobacter pylori* Infection on Intestinal Microbiota, Immunity and Colorectal Cancer Risk. Front. Cell Infect. Microbiol..

[4] Engstrand L., Lindberg M. (2013). *Helicobacter pylori* and the Gastric Microbiota. Best Pract. Res. Clin. Gastroenterol..

[5] Rueda-Robles A., Rubio-Tomás T., Plaza-Diaz J., Álvarez-Mercado A.I. (2021). Impact of Dietary Patterns on *H. pylori* Infection and the Modulation of Microbiota to Counteract Its Effect. A Narrative Review. Pathogens.

[6] Naumann M., Ferino L., Sharafutdinov I., Backert S. (2023). Gastric Epithelial Barrier Disruption, Inflammation and Oncogenic Signal Transduction by *Helicobacter pylori*. Curr. Top. Microbiol. Immunol..

[7] Hatakeyama M. (2023). Impact of the *Helicobacter pylori* Oncoprotein CagA in Gastric Carcinogenesis. Curr. Top. Microbiol. Immunol..

[8] Ohnishi N., Yuasa H., Tanaka S., Sawa H., Miura M., Matsui A., Higashi H., Musashi M., Iwabuchi K., Suzuki M. (2008). Transgenic Expression of *Helicobacter pylori* CagA Induces Gastrointestinal and Hematopoietic Neoplasms in Mouse. Proc. Natl. Acad. Sci. USA.

[9] Botham C.M., Wandler A.M., Guillemin K. (2008). A Transgenic Drosophila Model Demonstrates That the *Helicobacter pylori* CagA Protein Functions as a Eukaryotic Gab Adaptor. PLoS Pathog..

[10] Neal J.T., Peterson T.S., Kent M.L., Guillemin K. (2013). *H. pylori* Virulence Factor CagA Increases Intestinal Cell Proliferation by Wnt Pathway Activation in a Transgenic Zebrafish Model. Dis. Model. Mech..

[11] Chauhan N., Tay A.C.Y., Marshall B.J., Jain U. (2019). *Helicobacter pylori* VacA, a Distinct Toxin Exerts Diverse Functionalities in Numerous Cells: An Overview. Helicobacter.

[12] Blaser N., Backert S., Pachathundikandi S.K. (2019). Immune Cell Signaling by *Helicobacter pylori*: Impact on Gastric Pathology. Adv. Exp. Med. Biol..

[13] Pachathundikandi S.K., Tegtmeyer N., Backert S. (2013). Signal Transduction of *Helicobacter pylori* during Interaction with Host Cell Protein Receptors of Epithelial and Immune Cells. Gut Microbes.

[14] Tegtmeyer N., Neddermann M., Asche C.I., Backert S. (2017). Subversion of Host Kinases: A Key Network in Cellular Signaling Hijacked by *Helicobacter pylori* CagA. Mol. Microbiol..

[15] Sharafutdinov I., Tegtmeyer N., Linz B., Rohde M., Vieth M., Tay A.C.-Y., Lamichhane B., Tuan V.P., Fauzia K.A., Sticht H. (2023). A Single-Nucleotide Polymorphism in *Helicobacter pylori* Promotes Gastric Cancer Development. Cell Host Microbe.

[16] Ansari S., Yamaoka Y. (2019). Helicobacter Pylori Virulence Factors Exploiting Gastric Colonization and Its Pathogenicity. Toxins.

[17] Roos S., Engstrand L., Jonsson H. (2005). Lactobacillus gastricus Sp. Nov., Lactobacillus antri Sp. Nov., Lactobacillus kalixensis Sp. Nov. and Lactobacillus ultunensis Sp. Nov., Isolated from Human Stomach Mucosa. Int. J. Syst. Evol. Microbiol..

[18] Ruggiero P. (2014). Use of Probiotics in the Fight against *Helicobacter pylori*. World J. Gastrointest. Pathophysiol..

[19] Abid S., Farid A., Abid R., Rehman M., Alsanie W., Alhomrani M., Alamri A., Asdaq S., Hefft D., Saqib S. (2022). Identification, Biochemical Characterization, and Safety Attributes of Locally Isolated *Lactobacillus fermentum* from *Bubalus bubalis* (Buffalo) Milk as a Probiotic. Microorganisms.

[20] Qureshi N., Li P., Gu Q. (2019). Probiotic Therapy in Helicobacter Pylori Infection: A Potential Strategy against a Serious Pathogen?. Appl. Microbiol. Biotechnol..

[21] Noto J.M., Peek R.M. (2017). The Gastric Microbiome, Its Interaction with *Helicobacter pylori*, and Its Potential Role in the Progression to Stomach Cancer. PLoS Pathog..

[22] Yang H., Hu B. (2022). Immunological Perspective: *Helicobacter pylori* Infection and Gastritis. Mediat. Inflamm..

[23] Malfertheiner P., Camargo M.C., El-Omar E., Liou J.-M., Peek R., Schulz C., Smith S.I., Suerbaum S. (2023). *Helicobacter pylori* Infection. Nat. Rev. Dis. Primers.

[24] Cover T.L., Lacy D.B., Ohi M.D. (2020). The *Helicobacter pylori* Cag Type IV Secretion System. Trends Microbiol..

[25] Pachathundikandi S.K., Tegtmeyer N., Arnold I.C., Lind J., Neddermann M., Falkeis-Veits C., Chattopadhyay S., Brönstrup M., Tegge W., Hong M. (2019). T4SS-Dependent TLR5 Activation by *Helicobacter pylori* Infection. Nat. Commun..

[26] Tegtmeyer N., Neddermann M., Lind J., Pachathundikandi S.K., Sharafutdinov I., Gutiérrez-Escobar A.J., Brönstrup M., Tegge W., Hong M., Rohde M. (2020). Toll-like Receptor 5 Activation by the CagY Repeat Domains of *Helicobacter pylori*. Cell Rep..

[27] Nakano M., Yahiro K., Yamasaki E., Kurazono H., Akada J., Yamaoka Y., Niidome T., Hatakeyama M., Suzuki H., Yamamoto T. (2016). *Helicobacter pylori* VacA, Acting through Receptor Protein Tyrosine Phosphatase α, Is Crucial for CagA Phosphorylation in Human Duodenum Carcinoma Cell Line AZ-521. Dis. Models Mech..

[28] Sewald X., Gebert-Vogl B., Prassl S., Barwig I., Weiss E., Fabbri M., Osicka R., Schiemann M., Busch D.H., Semmrich M. (2008). Integrin Subunit CD18 Is the T-Lymphocyte Receptor for the *Helicobacter pylori* Vacuolating Cytotoxin. Cell Host Microbe.

[29] Pachathundikandi S.K., Blaser N., Backert S., Backert S. (2019). Mechanisms of Inflammasome Signaling, microRNA Induction and Resolution of Inflammation by Helicobacter Pylori. Molecular Mechanisms of Inflammation: Induction, Resolution and Escape by Helicobacter pylori.

[30] Pachathundikandi K., Backert S. (2018). Heptose 1,7-Bisphosphate Directed TIFA Oligomerization: A Novel PAMP-Recognizing Signaling Platform in the Control of Bacterial Infections. Gastroenterology.

[31] Pachathundikandi S.K., Tegtmeyer N., Backert S. (2023). Masking of Typical TLR4 and TLR5 Ligands Modulates Inflammation and Resolution by *Helicobacter pylori*. Trends Microbiol..

[32] Sokolova O., Maubach G., Naumann M. (2023). *Helicobacter pylori* Regulates TIFA Turnover in Gastric Epithelial Cells. Eur. J. Cell Biol..

[33] Alexander S.M., Retnakumar R.J., Chouhan D., Devi T.N.B., Dharmaseelan S., Devadas K., Thapa N., Tamang J.P., Lamtha S.C., Chattopadhyay S. (2021). *Helicobacter pylori* in Human Stomach: The Inconsistencies in Clinical Outcomes and the Probable Causes. Front. Microbiol..

[34] Moodley Y., Linz B., Bond R.P., Nieuwoudt M., Soodyall H., Schlebusch C.M., Bernhöft S., Hale J., Suerbaum S., Mugisha L. (2012). Age of the Association between *Helicobacter pylori* and Man. PLoS Pathog..

[35] Linz B., Balloux F., Moodley Y., Manica A., Liu H., Roumagnac P., Falush D., Stamer C., Prugnolle F., Van Der Merwe S.W. (2007). An African Origin for the Intimate Association between Humans and *Helicobacter pylori*. Nature.

[36] Moodley Y., Brunelli A., Ghirotto S., Klyubin A., Maady A.S., Tyne W., Muñoz-Ramirez Z.Y., Zhou Z., Manica A., Linz B. (2021). *Helicobacter pylori*’s Historical Journey through Siberia and the Americas. Proc. Natl. Acad. Sci. USA.

[37] Nath A.N., Retnakumar R.J., Francis A., Chhetri P., Thapa N., Chattopadhyay S. (2022). Peptic Ulcer and Gastric Cancer: Is It All in the Complex Host–Microbiome Interplay That Is Encoded in the Genomes of “Us” and “Them”?. Front. Microbiol..

[38] Backert S., Tegtmeyer N., Selbach M. (2010). The Versatility of *Helicobacter pylori* CagA Effector Protein Functions: The Master Key Hypothesis. Helicobacter.

[39] Chang W.-L., Yeh Y.-C., Sheu B.-S. (2018). The Impacts of *H. pylori* Virulence Factors on the Development of Gastroduodenal Diseases. J. Biomed. Sci..

[40] Rawla P., Barsouk A. (2019). Epidemiology of Gastric Cancer: Global Trends, Risk Factors and Prevention. Gastroenterol. Rev..

[41] Backert S., Haas R., Gerhard M., Naumann M. (2017). The *Helicobacter pylori* Type IV Secretion System Encoded by the Cag Pathogenicity Island: Architecture, Function, and Signaling. Curr. Top. Microbiol. Immunol..

[42] Hatakeyama M. (2014). *Helicobacter pylori* CagA and Gastric Cancer: A Paradigm for Hit-and-Run Carcinogenesis. Cell Host Microbe.

[43] Argent R.H., Thomas R.J., Letley D.P., Rittig M.G., Hardie K.R., Atherton J.C. (2008). Functional Association between the *Helicobacter pylori* Virulence Factors VacA and CagA. J. Med. Microbiol..

[44] Tegtmeyer N., Zabler D., Schmidt D., Hartig R., Brandt S., Backert S. (2009). Importance of EGF Receptor, HER2/Neu and Erk1/2 Kinase Signalling for Host Cell Elongation and Scattering Induced by the *Helicobacter pylori* CagA Protein: Antagonistic Effects of the Vacuolating Cytotoxin VacA. Cell Microbiol..

[45] Aydın E.M., Demir T.D., Seymen N., Said S.S., Oktem-Okullu S., Tiftikci A., Cicek B., Tokat F., Tozun N., Ince U. (2021). The Crosstalk between *H. pylori* Virulence Factors and the PD1:PD-L1 Immune Checkpoint Inhibitors in Progression to Gastric Cancer. Immunol. Lett..

[46] Zarzecka U., Tegtmeyer N., Sticht H., Backert S. (2023). Trimer Stability of *Helicobacter pylori* HtrA Is Regulated by a Natural Mutation in the Protease Domain. Med. Microbiol. Immunol..

[47] Thursby E., Juge N. (2017). Introduction to the Human Gut Microbiota. Biochem. J..

[48] Hugon P., Dufour J.-C., Colson P., Fournier P.-E., Sallah K., Raoult D. (2015). A Comprehensive Repertoire of Prokaryotic Species Identified in Human Beings. Lancet Infect. Dis..

[49] Almeida A., Mitchell A.L., Boland M., Forster S.C., Gloor G.B., Tarkowska A., Lawley T.D., Finn R.D. (2019). A New Genomic Blueprint of the Human Gut Microbiota. Nature.

[50] Syromyatnikov M., Nesterova E., Gladkikh M., Smirnova Y., Gryaznova M., Popov V. (2022). Characteristics of the Gut Bacterial Composition in People of Different Nationalities and Religions. Microorganisms.

[51] Tremaroli V., Bäckhed F. (2012). Functional Interactions between the Gut Microbiota and Host Metabolism. Nature.

[52] Wu W.M., Yang Y.S., Peng L.H. (2014). Microbiota in the Stomach: New Insights. J. Dig. Dis..

[53] Park J.Y., Seo H., Kang C.-S., Shin T.-S., Kim J.W., Park J.-M., Kim J.G., Kim Y.-K. (2022). Dysbiotic Change in Gastric Microbiome and Its Functional Implication in Gastric Carcinogenesis. Sci. Rep..

[54] Rinninella E., Raoul P., Cintoni M., Franceschi F., Miggiano G.A.D., Gasbarrini A., Mele M.C. (2019). What Is the Healthy Gut Microbiota Composition? A Changing Ecosystem across Age, Environment, Diet, and Diseases. Microorganisms.

[55] Bik E.M., Eckburg P.B., Gill S.R., Nelson K.E., Purdom E.A., Francois F., Perez-Perez G., Blaser M.J., Relman D.A. (2006). Molecular Analysis of the Bacterial Microbiota in the Human Stomach. Proc. Natl. Acad. Sci. USA.

[56] Hsieh Y.-Y., Tung S.-Y., Pan H.-Y., Yen C.-W., Xu H.-W., Lin Y.-J., Deng Y.-F., Hsu W.-T., Wu C.-S., Li C. (2018). Increased Abundance of Clostridium and Fusobacterium in Gastric Microbiota of Patients with Gastric Cancer in Taiwan. Sci. Rep..

[57] Andersson A.F., Lindberg M., Jakobsson H., Bäckhed F., Nyrén P., Engstrand L. (2008). Comparative Analysis of Human Gut Microbiota by Barcoded Pyrosequencing. PLoS ONE.

[58] Linz B., Backert S. (2021). *Helicobacter pylori* and the Gut Microbiota. Microbiota Health Dis..

[59] Auchtung T.A., Fofanova T.Y., Stewart C.J., Nash A.K., Wong M.C., Gesell J.R., Auchtung J.M., Ajami N.J., Petrosino J.F. (2018). Investigating Colonization of the Healthy Adult Gastrointestinal Tract by Fungi. mSphere.

[60] Wong J.M. (2014). Gut Microbiota and Cardiometabolic Outcomes: Influence of Dietary Patterns and Their Associated Components. Am. J. Clin. Nutr..

[61] Hasan N., Yang H. (2019). Factors Affecting the Composition of the Gut Microbiota, and Its Modulation. PeerJ.

[62] Serban D.E. (2011). The Gut Microbiota in the Metagenomics Era: Sometimes a Friend, Sometimes a Foe. Roum. Arch. Microbiol. Immunol..

[63] Lehr K., Nikitina D., Vilchez-Vargas R., Steponaitiene R., Thon C., Skieceviciene J., Schanze D., Zenker M., Malfertheiner P., Kupcinskas J. (2023). Microbial Composition of Tumorous and Adjacent Gastric Tissue Is Associated with Prognosis of Gastric Cancer. Sci. Rep..

[64] Rolig A.S., Cech C., Ahler E., Carter J.E., Ottemann K.M. (2013). The Degree of *Helicobacter pylori*-Triggered Inflammation Is Manipulated by Preinfection Host Microbiota. Infect. Immun..

[65] Nasrollahzadeh D., Malekzadeh R., Ploner A., Shakeri R., Sotoudeh M., Fahimi S., Nasseri-Moghaddam S., Kamangar F., Abnet C.C., Winckler B. (2015). Variations of Gastric Corpus Microbiota Are Associated with Early Esophageal Squamous Cell Carcinoma and Squamous Dysplasia. Sci. Rep..

[66] Li T.H., Qin Y., Sham P.C., Lau K.S., Chu K.-M., Leung W.K. (2017). Alterations in Gastric Microbiota After *H. pylori* Eradication and in Different Histological Stages of Gastric Carcinogenesis. Sci. Rep..

[67] Devi T.B., Devadas K., George M., Gandhimathi A., Chouhan D., Retnakumar R.J., Alexander S.M., Varghese J., Dharmaseelan S., Chandrika S.K. (2021). Low Bifidobacterium Abundance in the Lower Gut Microbiota Is Associated with *Helicobacter pylori*-Related Gastric Ulcer and Gastric Cancer. Front. Microbiol..

[68] Durán C., Ciucci S., Palladini A., Ijaz U.Z., Zippo A.G., Sterbini F.P., Masucci L., Cammarota G., Ianiro G., Spuul P. (2021). Nonlinear Machine Learning Pattern Recognition and Bacteria-Metabolite Multilayer Network Analysis of Perturbed Gastric Microbiome. Nat. Commun..

[69] Ianiro G., Molina-Infante J., Gasbarrini A. (2015). Gastric Microbiota. Helicobacter.

[70] Guo Y., Zhang Y., Gerhard M., Gao J.-J., Mejias-Luque R., Zhang L., Vieth M., Ma J.-L., Bajbouj M., Suchanek S. (2020). Effect of *Helicobacter pylori* on Gastrointestinal Microbiota: A Population-Based Study in Linqu, a High-Risk Area of Gastric Cancer. Gut.

[71] Miftahussurur M., Waskito L.A., El-Serag H.B., Ajami N.J., Nusi I.A., Syam A.F., Matsumoto T., Rezkitha Y.A.A., Doohan D., Fauzia K.A. (2020). Gastric Microbiota and *Helicobacter pylori* in Indonesian Population. Helicobacter.

[72] Ndegwa N., Ploner A., Andersson A.F., Zagai U., Andreasson A., Vieth M., Talley N.J., Agreus L., Ye W. (2020). Gastric Microbiota in a Low-*Helicobacter pylori* Prevalence General Population and Their Associations with Gastric Lesions. Clin. Transl. Gastroenterol..

[73] Kadeerhan G., Gerhard M., Gao J.-J., Mejías-Luque R., Zhang L., Vieth M., Ma J.-L., Bajbouj M., Suchanek S., Liu W.-D. (2021). Microbiota Alteration at Different Stages in Gastric Lesion Progression: A Population-Based Study in Linqu, China. Am. J. Cancer Res..

[74] Jo H.J., Kim J., Kim N., Park J.H., Nam R.H., Seok Y.-J., Kim Y.-R., Kim J.S., Kim J.M., Kim J.M. (2016). Analysis of Gastric Microbiota by Pyrosequencing: Minor Role of Bacteria Other than *Helicobacter pylori* in the Gastric Carcinogenesis. Helicobacter.

[75] Rajilic-Stojanovic M., Figueiredo C., Smet A., Hansen R., Kupcinskas J., Rokkas T., Andersen L., Machado J.C., Ianiro G., Gasbarrini A. (2020). Systematic Review: Gastric Microbiota in Health and Disease. Aliment. Pharmacol. Ther..

[76] Barra W.F., Sarquis D.P., Khayat A.S., Khayat B.C.M., Demachki S., Anaissi A.K.M., Ishak G., Santos N.P.C., Dos Santos S.E.B., Burbano R.R. (2021). Gastric Cancer Microbiome. Pathobiology.

[77] Pereira-Marques J., Ferreira R.M., Machado J.C., Figueiredo C. (2021). The Influence of the Gastric Microbiota in Gastric Cancer Development. Best. Pract. Res. Clin. Gastroenterol..

[78] Shen Z., Dzink-Fox J., Feng Y., Muthupalani S., Mannion A.J., Sheh A., Whary M.T., Holcombe H.R., Piazuelo B.M., Bravo L.E. (2022). Gastric Non-*Helicobacter pylori* Urease-Positive Staphylococcus Epidermidis and Streptococcus Salivarius Isolated from Humans Have Contrasting Effects on *H. pylori*-Associated Gastric Pathology and Host Immune Responses in a Murine Model of Gastric Cancer. mSphere.

[79] Zilberstein B., Quintanilha A.G., Santos M.A.A., Pajecki D., Moura E.G., Alves P.R.A., Filho F.M., Souza J.A.U.D., Gama-Rodrigues J. (2007). Digestive Tract Microbiota In Healthy Volunteers. Clinics.

[80] Dicksved J., Lindberg M., Rosenquist M., Enroth H., Jansson J.K., Engstrand L. (2009). Molecular Characterization of the Stomach Microbiota in Patients with Gastric Cancer and in Controls. J. Med. Microbiol..

[81] Mărginean C.O., Meliț L.E., Săsăran M.O. (2021). Gastric Microenvironment—A Partnership between Innate Immunity and Gastric Microbiota Tricks *Helicobacter pylori*. J. Clin. Med..

[82] Zaman C., Osaki T., Hanawa T., Yonezawa H., Kurata S., Kamiya S. (2014). Analysis of the Microbial Ecology between *Helicobacter pylori* and the Gastric Microbiota of Mongolian Gerbils. J. Med. Microbiol..

[83] Delgado S., Leite A.M.O., Ruas-Madiedo P., Mayo B. (2015). Probiotic and Technological Properties of *Lactobacillus* Spp. Strains from the Human Stomach in the Search for Potential Candidates against Gastric Microbial Dysbiosis. Front. Microbiol..

[84] Pimentel-Nunes P., Gonçalves N., Boal-Carvalho I., Afonso L., Lopes P., Roncon-Albuquerque R., Henrique R., Moreira-Dias L., Leite-Moreira A.F., Dinis-Ribeiro M. (2013). *Helicobacter pylori* Induces Increased Expression of Toll-Like Receptors and Decreased Toll-Interacting Protein in Gastric Mucosa That Persists Throughout Gastric Carcinogenesis. Helicobacter.

[85] Aviles-Jimenez F., Vazquez-Jimenez F., Medrano-Guzman R., Mantilla A., Torres J. (2014). Stomach Microbiota Composition Varies between Patients with Non-Atrophic Gastritis and Patients with Intestinal Type of Gastric Cancer. Sci. Rep..

[86] Kachuei V., Talebi Bezmin Abadi A., Rahimi F., Forootan M. (2020). Colonization by Pseudomonas Aeruginosa and Staphylococcus Aureus of Antral Biopsy Specimens from Gastritis Patients Uninfected with *Helicobacter pylori*. Infect. Drug Resist..

[87] Sanchez-Villamil J.I., Tapia D., Borlee G.I., Borlee B.R., Walker D.H., Torres A.G. (2020). *Burkholderia pseudomallei* as an Enteric Pathogen: Identification of Virulence Factors Mediating Gastrointestinal Infection. Infect. Immun..

[88] Khosravi Y., Dieye Y., Loke M.F., Goh K.L., Vadivelu J. (2014). *Streptococcus mitis* Induces Conversion of *Helicobacter pylori* to Coccoid Cells during Co-Culture In Vitro. PLoS ONE.

[89] Heimesaat M.M., Fischer A., Plickert R., Wiedemann T., Loddenkemper C., Göbel U.B., Bereswill S., Rieder G. (2014). *Helicobacter pylori* Induced Gastric Immunopathology Is Associated with Distinct Microbiota Changes in the Large Intestines of Long-Term Infected Mongolian Gerbils. PLoS ONE.

[90] Das A., Pereira V., Saxena S., Ghosh T.S., Anbumani D., Bag S., Das B., Nair G.B., Abraham P., Mande S.S. (2017). Gastric Microbiome of Indian Patients with *Helicobacter pylori* Infection, and Their Interaction Networks. Sci. Rep..

[91] Zheng W., Miao J., Luo L., Long G., Chen B., Shu X., Gu W., Peng K., Li F., Zhao H. (2021). The Effects of *Helicobacter pylori* Infection on Microbiota Associated with Gastric Mucosa and Immune Factors in Children. Front. Immunol..

[92] Brawner K.M., Morrow C.D., Smith P.D. (2014). Gastric Microbiome and Gastric Cancer. Cancer J..

[93] Montalban-Arques A., Wurm P., Trajanoski S., Schauer S., Kienesberger S., Halwachs B., Högenauer C., Langner C., Gorkiewicz G. (2016). *Propionibacterium acnes* Overabundance and Natural Killer Group 2 Member D System Activation in Corpus-dominant Lymphocytic Gastritis. J. Pathol..

[94] Velickovic M., Arsenijevic A., Acovic A., Arsenijevic D., Milovanovic J., Dimitrijevic J., Todorovic Z., Milovanovic M., Kanjevac T., Arsenijevic N. (2021). Galectin-3, Possible Role in Pathogenesis of Periodontal Diseases and Potential Therapeutic Target. Front. Pharmacol..

[95] Park A.-M., Hagiwara S., Hsu D.K., Liu F.-T., Yoshie O. (2016). Galectin-3 Plays an Important Role in Innate Immunity to Gastric Infection by *Helicobacter pylori*. Infect. Immun..

[96] Klymiuk I., Bilgilier C., Stadlmann A., Thannesberger J., Kastner M.-T., Högenauer C., Püspök A., Biowski-Frotz S., Schrutka-Kölbl C., Thallinger G.G. (2017). The Human Gastric Microbiome Is Predicated upon Infection with *Helicobacter pylori*. Front. Microbiol..

[97] Chen R., Li Y., Chen X., Chen J., Song J., Yang X., Ye L., Wu Z., Xie P., Zhong Q. (2023). *dupA*^+^ *H. pylori* Reduces Diversity of Gastric Microbiome and Increases Risk of Erosive Gastritis. Front. Cell. Infect. Microbiol..

[98] Ge Z., Sheh A., Feng Y., Muthupalani S., Ge L., Wang C., Kurnick S., Mannion A., Whary M.T., Fox J.G. (2018). Helicobacter Pylori-Infected C57BL/6 Mice with Different Gastrointestinal Microbiota Have Contrasting Gastric Pathology, Microbial and Host Immune Responses. Sci. Rep..

[99] Shirasawa Y., Shibahara-Sone H., Iino T., Ishikawa F. (2010). Bifidobacterium Bifidum BF-1 Suppresses *Helicobacter pylori*-Induced Genes in Human Epithelial Cells. J. Dairy Sci..

[100] Saracino I.M., Pavoni M., Saccomanno L., Fiorini G., Pesci V., Foschi C., Piccirilli G., Bernardini G., Holton J., Figura N. (2020). Antimicrobial Efficacy of Five Probiotic Strains Against *Helicobacter pylori*. Antibiotics.

[101] Taghizadeh S., Falsafi T., Kermanshahi R.K., Ramezani R. (2020). Antagonistic and Immunomodulant Effects of Two Probiotic Strains of Lactobacillus on Clinical Strains of *Helicobacter pylori*. Galen. Med. J..

[102] Lin C.-C., Huang W.-C., Su C.-H., Lin W.-D., Wu W.-T., Yu B., Hsu Y.-M. (2020). Effects of Multi-Strain Probiotics on Immune Responses and Metabolic Balance in *Helicobacter pylori*-Infected Mice. Nutrients.

[103] Maleki-Kakelar H., Dehghani J., Barzegari A., Barar J., Shirmohamadi M., Sadeghi J., Omidi Y. (2020). Lactobacillus Plantarum Induces Apoptosis in Gastric Cancer Cells via Modulation of Signaling Pathways in *Helicobacter pylori*. Bioimpacts.

[104] Yarmohammadi M., Yadegar A., Ebrahimi M.T., Zali M.R. (2021). Effects of a Potential Probiotic Strain Lactobacillus Gasseri ATCC 33323 on *Helicobacter pylori*-Induced Inflammatory Response and Gene Expression in Coinfected Gastric Epithelial Cells. Probiotics Antimicro. Prot..

[105] He C., Peng C., Xu X., Li N., Ouyang Y., Zhu Y., Lu N. (2022). Probiotics Mitigate *Helicobacter pylori*-Induced Gastric Inflammation and Premalignant Lesions in INS-GAS Mice with the Modulation of Gastrointestinal Microbiota. Helicobacter.

[106] Lai C.-H., Lin T.-L., Huang M.-Z., Li S.-W., Wu H.-Y., Chiu Y.-F., Yang C.-Y., Chiu C.-H., Lai H.-C. (2022). Gut Commensal Parabacteroides Goldsteinii MTS01 Alters Gut Microbiota Composition and Reduces Cholesterol to Mitigate *Helicobacter pylori*-Induced Pathogenesis. Front. Immunol..

[107] Sakarya S., Gunay N. (2014). *Saccharomyces Boulardii* Expresses Neuraminidase Activity Selective for A2,3-linked Sialic Acid That Decreases *Helicobacter pylori* Adhesion to Host Cells. APMIS.

[108] Whiteside S.A., Mohiuddin M.M., Shlimon S., Chahal J., MacPherson C.W., Jass J., Tompkins T.A., Creuzenet C. (2021). In Vitro Framework to Assess the Anti-*Helicobacter pylori* Potential of Lactic Acid Bacteria Secretions as Alternatives to Antibiotics. Int. J. Mol. Sci..

[109] Boyanova L., Gergova G., Markovska R., Yordanov D., Mitov I. (2017). Bacteriocin-like Inhibitory Activities of Seven *Lactobacillus delbrueckii* Subsp. Bulgaricus Strains against Antibiotic Susceptible and Resistant *Helicobacter pylori* Strains. Lett. Appl. Microbiol..

[110] Song H., Zhou L., Liu D., Ge L., Li Y. (2019). Probiotic Effect on *Helicobacter pylori* Attachment and Inhibition of Inflammation in Human Gastric Epithelial Cells. Exp. Ther. Med..

[111] Thiraworawong T., Spinler J.K., Werawatganon D., Klaikeaw N., Venable S.F., Versalovic J., Tumwasorn S. (2014). Anti-Inflammatory Properties of Gastric-Derived Lactobacillus Plantarum XB7 in the Context of *Helicobacter pylori* Infection. Helicobacter.

[112] Afsahi A., Mahmoudi H., Ebrahimi A., Aeini Z., Esmaeili D. (2018). Evaluation of the Effect of Lactobacillus Planetarium Probiotics Produced from Broad Bean Seed in Prevention of *Helicobacter pylori* in Stomach Tissue of C57BL/6 Mice. J. Cancer Sci. Ther..

[113] Yang Z., Zhou Y., Han Z., He K., Zhang Y., Wu D., Chen H. (2024). The Effects of Probiotics Supplementation on *Helicobacter pylori* Standard Treatment: An Umbrella Review of Systematic Reviews with Meta-Analyses. Sci. Rep..

[114] Abreu M.T., Peek R.M. (2014). Gastrointestinal Malignancy and the Microbiome. Gastroenterology.

[115] Niikura R., Hayakawa Y., Nagata N., Miyoshi-Akiayama T., Miyabayashi K., Tsuboi M., Suzuki N., Hata M., Arai J., Kurokawa K. (2023). Non-*Helicobacter pylori* Gastric Microbiome Modulates Prooncogenic Responses and Is Associated with Gastric Cancer Risk. Gastro Hep Adv..

[116] Li Y., Hu Y., Zhan X., Song Y., Xu M., Wang S., Huang X., Xu Z.Z. (2023). Meta-Analysis Reveals *Helicobacter pylori* Mutual Exclusivity and Reproducible Gastric Microbiome Alterations during Gastric Carcinoma Progression. Gut Microbes.

[117] Waskito L.A., Rezkitha Y.A.A., Vilaichone R., Sugihartono T., Mustika S., Dewa Nyoman Wibawa I., Yamaoka Y., Miftahussurur M. (2022). The Role of Non-*Helicobacter pylori* Bacteria in the Pathogenesis of Gastroduodenal Diseases. Gut Pathog..

[118] Eun C.S., Kim B.K., Han D.S., Kim S.Y., Kim K.M., Choi B.Y., Song K.S., Kim Y.S., Kim J.F. (2014). Differences in Gastric Mucosal Microbiota Profiling in Patients with Chronic Gastritis, Intestinal Metaplasia, and Gastric Cancer Using Pyrosequencing Methods. Helicobacter.

[119] Tuerk E., Doss S., Polsley K. (2023). Peptic Ulcer Disease. Prim. Care.

[120] Cao X., Yang Y., Zhang Y., Ji R., Zhao X., Zheng W., Yang A. (2023). Impact *of Helicobacter pylori* on the Gastric Microbiome in Patients with Chronic Gastritis: A Systematic Review and Meta-Analysis Protocol. BMJ Open.

[121] Li L., Zhou X., Xiao S., Ye F., Zhang G. (2016). The Effect of *Helicobacter pylori* Eradication on the Gastrointestinal Microbiota in Patients with Duodenal Ulcer. J. Gastrointest. Liver Dis..

[122] Ozbey G., Hanafiah A., Sproston E. (2020). *Helicobacter pylori* Infection and Gastric Microbiota. Euroasian J. Hepato-Gastroenterol..

[123] Ernst P.B., Jin Y., Reyes V.E., Crowe S.E. (1994). The Role of the Local Immune Response in the Pathogenesis of Peptic Ulcer Formation. Scand. J. Gastroenterol..

[124] Alejandra M.G. (2023). Risk Factors for Developing Peptic Ulcer Disease. Int. J. Med. Sci. Clin. Res. Stud..

[125] John Umaru I. (2023). Peptic Ulcer Disease and Its Implications. Res. Gastric Manag. Hepatol..

[126] Dong Z., Yu K., Xin Y., Gao X., Bu F., Zhao D., Ren D., Lu J., Wang D. (2024). Association between Gut Microbiota and Peptic Ulcer Disease, Particularly Gastric Ulcer and Duodenal Ulcer: A Two-Sample Mendelian Randomization Study. Front. Microbiol..

[127] Chen X., Xia C., Li Q., Jin L., Zheng L., Wu Z. (2018). Comparisons Between Bacterial Communities in Mucosa in Patients with Gastric Antrum Ulcer and a Duodenal Ulcer. Front. Cell. Infect. Microbiol..

[128] Boltin D. (2016). Probiotics in *Helicobacter pylori*-Induced Peptic Ulcer Disease. Best Pract. Res. Clin. Gastroenterol..

